# Sugarcane–peanut intercropping improves rhizosphere fungal communities and metabolites following long-term continuous peanut monoculture

**DOI:** 10.3389/fmicb.2026.1957778

**Published:** 2026-10-23

**Authors:** Ziqin Pang, Manqian Cha, Yongxin Lin, Xiumei Tang, Yongmei Zhou, Zhaonian Yuan, Jiapan Lian, Ronghua Tang, Tao Wang, Qi Zhang, Antonio Francesco Logrieco, Xiao Wang, Peiwu Li

**Affiliations:** 1 Xianghu Laboratory, Zhejiang Provincial Laboratory of Agriculture, Hangzhou, China; 2 National Engineering Research Center for Sugarcane, Fujian Agriculture and Forestry University, Fuzhou, China; 3 School of Geographical Sciences, Fujian Normal University, Fuzhou, China; 4 Cash Crops Research Institute, Guangxi Academy of Agricultural Sciences, Nanning, China; 5 State Key Laboratory for Conservation and Utilization of Subtropical Agri-Biological Resources, Guangxi University, Nanning, China; 6 Key Laboratory of Biology and Genetic Improvement of Oil Crops, Key Laboratory of Detection for Mycotoxins, Ministry of Agriculture and Rural Affairs, Oil Crops Research Institute, Chinese Academy of Agricultural Sciences, Wuhan, China

**Keywords:** continuous peanut monoculture, fungal community, rhizosphere microecology, row spacing, sugarcane–peanut intercropping, *Trichoderma*, untargeted metabolomics

## Abstract

**Background:**

Sugarcane-peanut intercropping improves land use efficiency and crop productivity, yet the rhizosphere mechanisms through which this system alleviates continuous peanut monoculture soil sickness and regulates rhizosphere fungal-metabolite balance remain poorly understood.

**Methods:**

This study integrated amplicon sequencing and untargeted metabolomics to investigate fungal community dynamics and metabolite profiles in the peanut rhizosphere under monoculture and three intercropping row spacings (2.0 m, 2.4 m, and 3.0 m).

**Results:**

Intercropping produced coordinated, row-spacing-dependent shifts in peanut rhizosphere fungal taxa, metabolite profiles, land-use efficiency, and kernel quality. Although overall fungal and metabolite beta-diversity differences were not significant, SP2.4 showed the largest set of differentially abundant fungal taxa, while SP2.0 achieved the highest land equivalent ratio. Across spacings, intercropping depleted several *Aspergillus* and *Fusarium* taxa and enriched candidate beneficial fungi, including *Trichoderma* species. Untargeted metabolomics further revealed spacing-specific enrichment of phenolic, stilbenoid, and terpenoid compounds with reported roles in plant defense or fungal inhibition. A feature putatively annotated as aflatoxin B_1_ by untargeted LC–MS/MS library matching was less abundant under intercropping. Together, the results indicate that sugarcane–peanut intercropping can improve the rhizosphere microecological state of long-term monocropped peanut fields through coordinated fungal and metabolic reorganization. A row-spacing window of 2.0–2.4 m provided the most favorable balance between system productivity and belowground microecological improvement.

## 1 Introduction

Peanut (*Arachis hypogaea* L.) is an important oilseed and food-legume crop that provides edible oil, protein, vitamins, and other nutrients for human consumption (Akhtar et al., 2014). However, repeated peanut monocropping can lead to soil sickness characterized by nutrient imbalance, deterioration of soil physicochemical properties, shifts in rhizosphere microbial communities, accumulation of soilborne pathogens, and declining crop productivity and quality (Li et al., 2014). Long-term peanut monocropping can disrupt the balance between potentially pathogenic and plant-beneficial fungal groups (Li et al., 2014). Peanut-field soil can also serve as a reservoir of *Aspergillus* and other soilborne fungi (Zhang et al., 2017). Restoring the rhizosphere microecological balance of long-term monocropped fields is therefore a central challenge for the sustainable intensification of peanut production. Diversified cropping systems may help restore degraded rhizosphere microecology by altering resource availability, root-derived chemical inputs, and microbial interactions.

Sugarcane–peanut intercropping is widely practiced in southern China and other subtropical regions and has received increasing attention because of its potential to improve land-use efficiency, crop productivity, soil fertility, and agroecosystem functioning (Tang et al., 2021; Pang et al., 2022; Fallah et al., 2026). Quantitatively, sugarcane–peanut intercropping achieved land equivalent ratios of 1.42 and 1.21 across two field sites, while the total economic return of the intercropping system was 87.84 and 36.38% higher than those of peanut and sugarcane monocultures, respectively (Tang et al., 2021; Fallah et al., 2026). Previous studies have shown that sugarcane–peanut intercropping improves soil physicochemical properties, alters rhizosphere microbial communities and metabolite profiles, and increases the abundance of potentially beneficial bacteria (Tang et al., 2021; Pang et al., 2022; Tang et al., 2022). At the microbial level, intercropping increased bacterial richness in sugarcane rhizosphere and bulk soils by 13.80 and 9.28%, respectively, relative to sugarcane monoculture, while more recent evidence showed selective enrichment of plant growth- and stress-regulating bacterial taxa under intercropping (Pang et al., 2022; Fallah et al., 2026). Our previous work further demonstrated that this intercropping system promotes crop health by recruiting beneficial bacteria and improving soil and crop productivity (Fallah et al., 2026), whereas a recent field comparison of long-term continuously cropped sugarcane showed that severely acidified soil (pH 3.47), compared with a nearby less-acidified sugarcane field (pH 5.35), was associated with reductions in flavonoids and other key metabolites in sugarcane roots and rhizosphere soil and with severe yield decline (Pang et al., 2025). Previous field studies have shown that sugarcane–peanut intercropping can increase soil pH relative to monocropping (Tang et al., 2021; Pang et al., 2022). One possible contributor is the additional application of calcium–magnesium phosphate fertilizer to peanut in the intercropping system, which supplies base cations such as Ca^2^ and Mg^2^ (Pang et al., 2022). Because the depletion of base cations is an important driver of soil acidification in subtropical sugarcane fields, their replenishment may partially explain the higher soil pH observed under intercropping (Pang et al., 2025). Sugarcane–peanut intercropping has also been shown to alter organic acids, soluble sugars, and phenolic acids in root exudates and to modify rhizosphere nutrient availability (Tang et al., 2022; Tang et al., 2024). However, its regulatory effects on the disordered peanut rhizosphere fungal community and the core metabolic feedback mechanisms that relieve monoculture soil sickness remain poorly understood.

The rhizosphere is a biologically and chemically active interface in which plant roots, soil, and microorganisms interact to regulate nutrient cycling, plant growth, and resistance to biotic and abiotic stresses (Philippot et al., 2013). Plant roots release a diverse range of primary and secondary metabolites that act as nutrients, signals, attractants, or antimicrobial compounds and thereby shape rhizosphere microbial-community assembly and function (Sasse et al., 2018; Zhalnina et al., 2018). Recent advances in high-throughput sequencing and mass-spectrometry-based metabolomics have enabled the simultaneous characterization of rhizosphere microbial communities and chemical profiles, providing new opportunities to identify coordinated microbiome–metabolite responses (van Dam and Bouwmeester, 2016; Pétriacq et al., 2017). Experimental studies have further demonstrated that root-derived metabolites can directly affect plant-associated organisms or indirectly enhance plant protection by restructuring the rhizosphere microbiota and recruiting pathogen-suppressive microorganisms (Hu et al., 2018; Liu et al., 2023; Zhou et al., 2023).

Plant-derived secondary metabolites may be particularly important in regulating rhizosphere fungal assembly and the proliferation of soilborne pathogens. Stilbenoids, phenolic acids, flavonoids, terpenoids, and fatty acids can act as chemical signals, antioxidants, or compounds that directly or indirectly suppress phytopathogens. For example, peanut-derived resveratrol derivatives and pinosylvin-related stilbenes inhibit the development of several *Aspergillus* species, while vanillic acid and other phenolics can interfere with fungal growth or cellular processes (Wang et al., 2015; Sobolev et al., 2017; Sobolev et al., 2019). Solavetivone and related sesquiterpenoid phytoalexins also exhibit activity against plant-pathogenic fungi (Harris and Dennis, 1976; Yokose et al., 2004). Fatty acids released into the rhizosphere may provide an additional layer of disease suppression; palmitic acid has been shown to inhibit the mycelial growth and sporulation of Fusarium oxysporum and to reduce Fusarium wilt severity, while more recent field evidence indicates that rhizosphere-secreted palmitic, oleic, linoleic, and stearic acids can enrich antagonistic Mortierella and suppress pathogenic *Fusarium* spp (Ma et al., 2021; Hu et al., 2026).

This study aimed to (1) characterize the effects of sugarcane–peanut intercropping on peanut rhizosphere fungal-community composition and diversity; (2) identify intercropping-responsive rhizosphere metabolites using untargeted metabolomics; and (3) compare three sugarcane row spacings to identify configurations that balance rhizosphere microecological improvement and crop productivity. We hypothesized that intercropping-associated changes in the rhizosphere chemical environment would enrich beneficial fungi and bioactive metabolites while reducing potentially pathogenic fungal groups, thereby alleviating the microecological deterioration associated with long-term peanut monocropping.

## 2 Materials and methods

### 2.1 Experimental design and field management

The field experiment was conducted at the sugarcane-peanut intercropping experimental site in Wuxuan County, Laibin City, Guangxi Zhuang Autonomous Region, China (109°50′-109°54′E, 23°48′-23°51′N), which has a subtropical monsoon climate. Before establishment of the experiment, the site had been continuously cropped with peanut for several consecutive years without crop rotation and was therefore representative of a long-term peanut monocropping field. The experiment comprised five cropping treatments (Figure 1): (1) peanut monocropping (PM, 1.0 m row spacing); (2) sugarcane monocropping (SM); and sugarcane-peanut intercropping with (3) 2.0 m (SP2.0), (4) 2.4 m (SP2.4), and (5) 3.0 m (SP3.0) sugarcane row spacings. Each treatment had three replicate plots arranged in a randomized complete block design (replicate plots are not shown in the schematic diagram in Figure 1). Plot areas were 72 m^2^ (PM, SM and SP2.4), 60 m^2^ (SP2.0) and 90 m^2^ (SP3.0).

**Figure 1:**
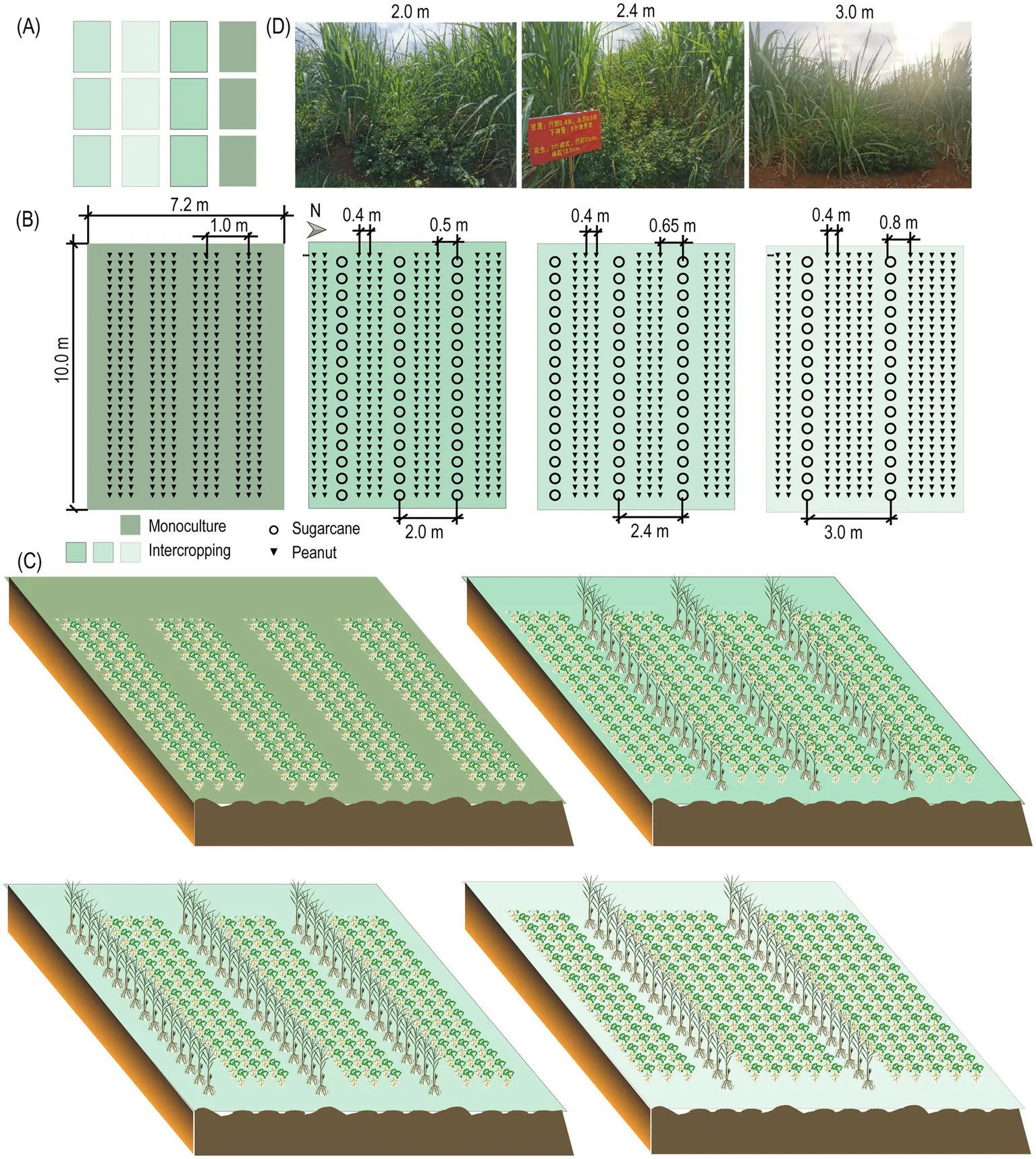
Schematic diagram of the field experimental layout. **(A)** Color-coded blocks showing the monoculture and intercropping treatments. **(B)** Two-dimensional schematic of the field layouts of peanut monocropping (PM, 1.0 m row spacing) and sugarcane–peanut intercropping with 2.0 m (SP2.0), 2.4 m (SP2.4) and 3.0 m (SP3.0) sugarcane row spacings; open circles indicate sugarcane and filled triangles indicate peanut. **(C)** Three-dimensional renderings illustrating the field arrangement of each treatment. **(D)** Photographs of the experimental fields at the 2.0, 2.4 and 3.0 m row spacings. Sugarcane monocropping (SM) was additionally maintained for land equivalent ratio calculation. Each treatment had three replicate plots arranged in a randomized complete block design (replicate plots are not shown in the schematic).

The sugarcane variety Yuetang 00/236 and the peanut variety Guihua 1,026 were used. Sugarcane was established directly in the field using double-bud stem setts, whereas peanut was directly sown in the field plots. In the intercropping treatments, three rows of sugarcane were alternated with nine rows of peanut at the 2.0 m and 2.4 m spacings, and with 15 rows of peanut at the 3.0 m spacing; the distance between adjacent sugarcane and peanut rows was 50, 65 and 80 cm for SP2.0, SP2.4 and SP3.0, respectively. Monocropped peanut was planted at a row spacing of 1.0 m. Sugarcane was fertilized with 525 kg/ha urea, 750 kg/ha calcium superphosphate and 300 kg/ha potassium chloride, while peanut received compound fertilizer (N: P_2_O_5_: K_2_O = 15:15:15) at 300 kg/ha as basal dressing. Irrigation and pest management followed standard local agricultural practices. The peanut growing period lasted approximately 122 days, from sowing on 10 March to harvest at physiological maturity on 10 July.

### 2.2 Peanut rhizosphere soil sampling

Because this study focused on the peanut crop, rhizosphere soil was collected from peanut plants at peanut harvest. Five peanut plants were randomly selected from each plot. Roots with adhering soil were shaken gently by hand for approximately 2 min to remove loosely adhering bulk soil, and the soil tightly attached to the root surface was then collected as rhizosphere soil with a sterile brush. Samples from the five plants were pooled to form one composite sample per plot, resulting in three replicate samples per treatment (12 samples in total for the four peanut treatments: PM, SP2.0, SP2.4 and SP3.0). Samples were immediately placed on ice and transported to the laboratory within 2 h. Upon arrival, each composite sample was divided into two portions: one stored at −80 °C for untargeted metabolomics and the other at −20 °C for DNA extraction.

### 2.3 Fungal ITS1 amplicon sequencing and bioinformatics

Total genomic DNA was extracted from the peanut rhizosphere soil samples. The fungal ITS1 region was amplified with the primer pair ITS1F (5′-CTTGGTCATTTAGAGGAAGTAA-3′) and ITS2 (5′-GCTGCGTTCTTCATCGATGC-3′) (White et al., 1990), with sequencing adapters attached; the expected amplicon size was approximately 300 bp. PCR products were purified, quantified and pooled at equal concentrations to construct sequencing libraries. After library quality control, qualified libraries were sequenced on the Illumina NovaSeq 6,000 platform (2 × 250 bp paired-end reads) at Biomarker Technologies Co., Ltd. (Beijing, China).

Raw reads were quality-filtered using Trimmomatic v0.33 (Bolger et al., 2014) using a 50-bp sliding window, trimming the 3′ end of reads when the average quality score within the window dropped below 20 (Phred scale). Primer sequences were identified and removed with cutadapt (v1.9.1; Martin, 2011), allowing a maximum mismatch rate of 20% and requiring at least 80% primer coverage. Quality-controlled reads were then denoised, merged, and de-chimerized using the DADA2 (Callahan et al., 2016) within QIIME2 (Bolyen et al., 2019) (truncQ = 2, maxEE = 2, minOverlap = 12 bp, maxMismatch = 0, and consensus chimera removal), thereby generating amplicon sequence variants (ASVs). Taxonomic annotation of ASVs was performed with a naive Bayes classifier against the UNITE Fungal database [Release 8.0; (Kõljalg et al., 2013)]. ASVs representing less than 0.005% of the total read count were discarded. To normalize sequencing depth, the fungal feature table was rarefied to an even depth of 73,394 sequences per sample. Species-level taxonomic assignments based on the ITS1 region should be considered putative owing to the variable resolution of ITS across fungal lineages; therefore, all biological interpretations in this study were made at the genus level or above.

Alpha diversity indices, including ACE, Chao1, Shannon, Simpson, PD whole tree and Good’s coverage, were calculated in QIIME2. Beta diversity was assessed based on Bray-Curtis and binary Jaccard distances and visualized by principal coordinate analysis (PCoA); the significance of community differentiation among treatments was tested by permutational multivariate analysis of variance (PERMANOVA, Adonis) using the vegan package in R. Differentially abundant fungal taxa between each intercropping treatment and peanut monoculture were identified using DESeq2 (Love et al., 2014). Pairwise differential-abundance analyses were performed for SP2.0 vs. PM, SP2.4 vs. PM, and SP3.0 vs. PM. *p* values were adjusted for multiple testing using the Benjamini–Hochberg false discovery rate (FDR) procedure, and taxa with FDR-adjusted *p* < 0.05 were considered differentially abundant. Differential taxa were visualized using Manhattan plots, in which enriched and depleted taxa are shown as upward and downward triangles, respectively. Venn diagrams were used to display shared and unique differential taxa among the three pairwise comparisons.

### 2.4 Untargeted metabolomics analysis

Metabolite profiling was performed on the peanut rhizosphere samples by LC–MS/MS. Rhizosphere soil samples were thawed on ice at 4 °C and homogenized. Approximately 100 mg of each soil sample was weighed into a centrifuge tube and mixed with 300 μL of methanol and 20 μL of internal standard. The mixture was vortexed for 30 s, subjected to ultrasonication for 10 min in ice water, and incubated for 1 h at −20 °C to precipitate proteins. After centrifugation at 13,000 rpm for 15 min at 4 °C, 200 μL of the supernatant was transferred into an LC–MS injection vial for analysis. Quality control (QC) samples were prepared by pooling 20 μL of the supernatant from each sample.

LC–MS/MS analysis was performed on a UHPLC system (1,290, Agilent Technologies) equipped with a UPLC BEH Amide column (1.7 μm, 2.1 × 100 mm, Waters) coupled to a TripleTOF 5,600 mass spectrometer (AB Sciex). The mobile phases consisted of 25 mM ammonium acetate and 25 mM ammonium hydroxide in water (pH 9.75) (A) and acetonitrile (B), delivered at 0.5 mL/min with the following gradient: 0 min, 95% B; 7 min, 65% B; 9 min, 40% B; 9.1 min, 95% B; 12 min, 95% B. The injection volume was 3 μL. The TripleTOF 5,600 was operated in information-dependent acquisition (IDA) mode controlled by Analyst TF 1.7 software (AB Sciex). In each cycle, 12 precursor ions with intensity greater than 100 were selected for fragmentation at a collision energy of 30 V, with 15 MS/MS events of 50 ms each. The electrospray ionization (ESI) source parameters were: ion source gas 1, 60 psi; ion source gas 2, 60 psi; curtain gas, 35 psi; source temperature, 650 °C; ion spray voltage floating, +5,000 V (positive mode) and −4,000 V (negative mode).

Raw MS data (.d files) were converted to mzXML format using ProteoWizard. Peak detection, extraction, alignment and integration were performed with the XCMS package (v3.2) in R, and peak annotation was conducted with the CAMERA package. Metabolite features were putatively annotated by matching against an in-house MS2 database and classified into chemical superclasses. Unless otherwise validated using authentic standards, metabolite identities were regarded as putative annotations. Differentially accumulated metabolites (DAMs) between each intercropping treatment and monoculture were identified using the combined criteria of |log2 fold change| ≥ 1 and Benjamini–Hochberg FDR-adjusted *p* < 0.05 and visualized with Manhattan plots; shared and unique differential metabolites among comparisons were displayed with Venn diagrams.

### 2.5 Agronomic trait measurement, quality analysis and land equivalent ratio

At harvest, peanut plants from each plot were sampled for measurement of agronomic traits, including total branch number, lateral branch length, pods per plant and filled pod rate. Peanut yield and sugarcane yield were determined from the plot harvest. Peanut kernel quality traits, including crude protein, crude fat and linoleic acid contents, were determined using near-infrared spectroscopy (NIRS). The land equivalent ratio (LER) was calculated as:


$$\mathrm{LER}=\frac{\mathrm{Yip}}{\mathrm{Ymp}}+\frac{\mathrm{Yis}}{\mathrm{Yms}}$$

Where Yip and Yis are the yields of intercropped peanut and sugarcane, and Ymp and Yms are the yields of monocropped peanut and sugarcane, respectively.

### 2.6 Statistical analysis

Statistical analyses were performed in R. Differences in agronomic traits, yield, quality parameters, and alpha diversity indices among treatments were tested by one-way analysis of variance (ANOVA) followed by Fisher’s least significant difference (LSD) test for multiple comparisons (*p* < 0.05). Beta diversity differences were tested by PERMANOVA (Adonis) with 999 permutations using the vegan package. Differentially abundant fungal taxa between PM and each intercropping treatment were identified using DESeq2 with Benjamini–Hochberg FDR-adjusted *p* < 0.05, as described in Section 2.3.

## 3 Result

### 3.1 Intercropping improves land use efficiency and peanut kernel quality

Sugarcane-peanut intercropping affected peanut agronomic performance and land use efficiency (Table 1; Figure 2). Total branch number, filled pod rate and pods per plant did not differ significantly among treatments (*p* > 0.05), whereas lateral branch length was significantly greater in SP2.0 than in PM and SP3.0 (*p* < 0.05), with SP2.4 intermediate. Peanut yield per hectare was highest in PM, followed by SP3.0, and was significantly lower in SP2.0 and SP2.4 owing to the reduced peanut planting density in intercropping (*p* < 0.05). In contrast, sugarcane yield in SP2.0 and SP2.4 was comparable to that of sugarcane monoculture (SM) and significantly higher than in SP3.0.

**Table 1:** Agronomic traits, yield, quality and land equivalent ratio (LER) of peanut and sugarcane under monoculture and intercropping treatments.

Item	PM	SP2.0	SP2.4	SP3.0	SM
Total branches (no.)	7.2a	6.8a	8.4a	6.8a	–
Lateral branch length (cm)	52.8b	58.6a	50.0ab	50.6b	–
Pods per plant (no.)	16.8a	17.8a	16.8a	18.0a	–
Filled pod rate (%)	78.9a	78.8a	81.6a	79.2a	–
Peanut yield (kg/ha)	3985a	2145c	2260c	3050b	–
Sugarcane yield (kg/ha)	–	92350a	82100a	61500b	98000a
LER peanut	1.00a	0.52c	0.55c	0.77b	–
LER sugarcane	–	0.96a	0.85a	0.65b	1.00a
LER total	1.00b	1.48a	1.40a	1.42a	1.00b

Values are means (*n* = 3). Different letters within columns indicate significant differences at *p* < 0.05 (one-way ANOVA followed by LSD test). Partial LER values were calculated against the corresponding monoculture (PM for peanut, SM for sugarcane). PM = peanut monocropping; SM = sugarcane monocropping; SP2.0, SP2.4, SP3.0 = sugarcane-peanut intercropping with 2.0, 2.4 and 3.0 m row spacings. Kernel quality traits (crude protein, crude fat and linoleic acid) are shown in Figures 2J–L.

**Figure 2:**
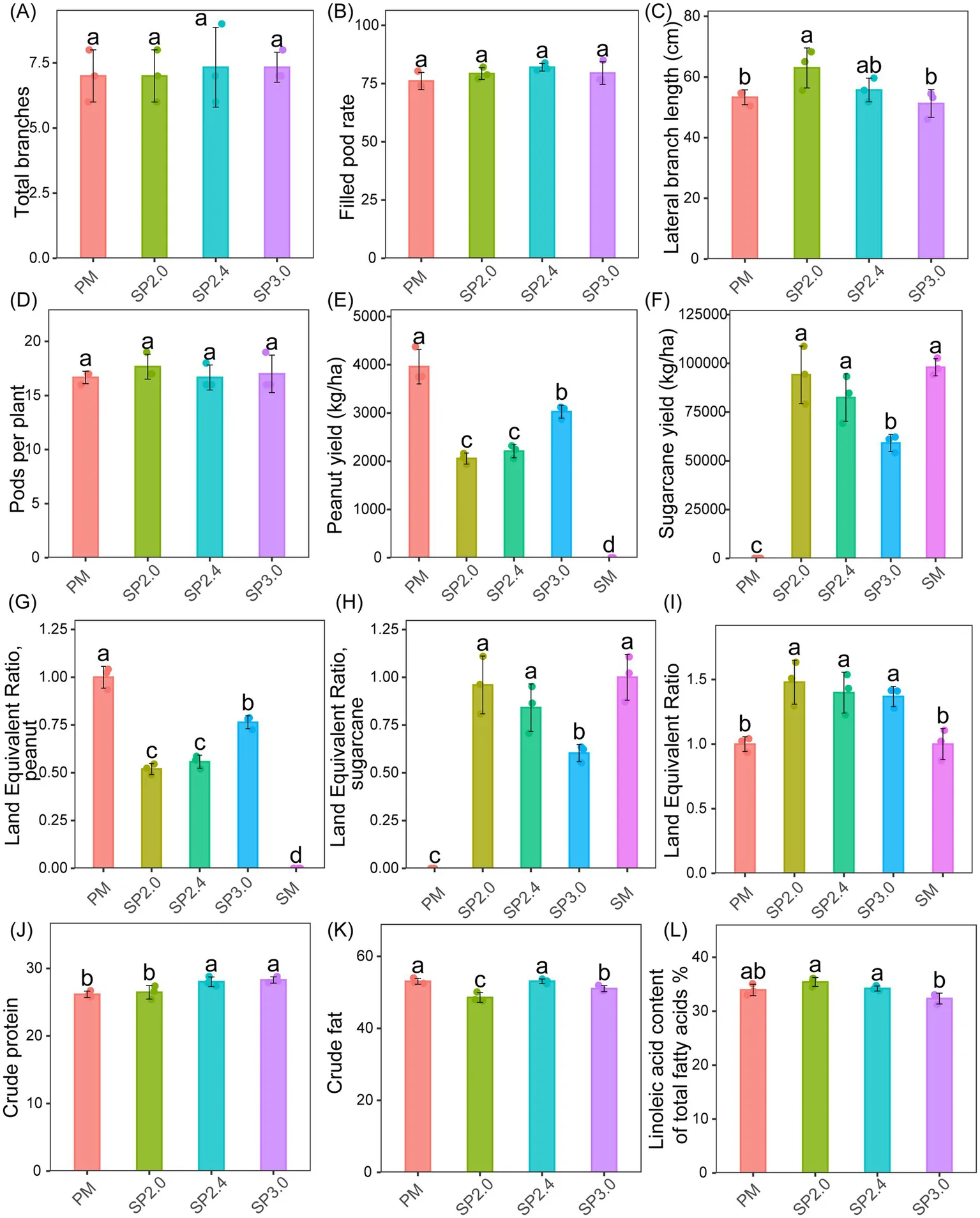
Agronomic traits, yield components, land equivalent ratio (LER) and kernel quality of peanut and sugarcane under monoculture and intercropping treatments. **(A)** Total branches; **(B)** Filled pod rate; **(C)** Lateral branch length; **(D)** Pods per plant; **(E)** Peanut yield; **(F)** Sugarcane yield; **(G)** Peanut partial LER; **(H)** Sugarcane partial LER; **(I)** Total LER; **(J)** Crude protein content; **(K)** Crude fat content; **(L)** Linoleic acid content. Different letters indicate significant differences at *p* < 0.05 (one-way ANOVA followed by LSD test; *n* = 3). PM = peanut monocropping; SM = sugarcane monocropping; SP2.0, SP2.4, SP3.0 = sugarcane-peanut intercropping with 2.0, 2.4 and 3.0 m row spacings.

Land equivalent ratio analysis showed that the peanut partial LER was highest in PM (1.00), followed by SP3.0 (0.77), and was lower in SP2.0 (0.52) and SP2.4 (0.55), whereas the sugarcane partial LER was highest in SP2.0 (0.96) and SP2.4 (0.85), which did not differ significantly from SM (1.00), and lowest in SP3.0 (0.65). Importantly, the total LER of all three intercropping treatments exceeded 1.0 and was significantly higher than that of the monocultures, with SP2.0 achieving the highest total LER (1.48), followed by SP3.0 (1.42) and SP2.4 (1.40). These results demonstrate a clear overyielding advantage of intercropping, with the greatest land use efficiency at the 2.0 m row spacing.

Kernel quality traits also responded to intercropping. Crude protein content was significantly higher in SP2.4 and SP3.0 than in PM and SP2.0. Crude fat content was highest in PM and SP2.4, intermediate in SP3.0, and lowest in SP2.0. Linoleic acid content was significantly higher in SP2.0 and SP2.4 than in SP3.0, with PM intermediate. These results indicate that intercropping at 2.0–2.4 m improves land productivity while maintaining or improving peanut kernel quality.

### 3.2 Intercropping reshapes peanut rhizosphere fungal community composition

ITS1 amplicon sequencing of the 12 peanut rhizosphere samples generated 959,354 raw paired-end reads. After quality filtering and primer removal, 955,780 clean reads remained. Denoising retained 955,322 reads, paired-end merging yielded 933,862 reads, and 930,004 non-chimeric reads remained after chimera removal, corresponding to an overall retention rate of 96.9% relative to the raw reads. The merged ITS1 sequence lengths were predominantly distributed between approximately 220 and 430 bp. Following taxonomic assignment against the UNITE fungal database, only ASVs assigned to the kingdom Fungi were retained for downstream fungal community analyses. The final fungal feature table contained 1,318 ASVs across the 12 samples. After rarefaction to an even depth of 73,394 sequences per sample, observed ASV richness ranged from 110 to 305 among individual samples (Supplementary Table S1).

PCoA based on Bray-Curtis distances showed that PM samples tended to separate from intercropping samples along the first two axes, which explained 27 and 20% of the total variation, respectively (Figure 3A). Although the community differentiation among treatments did not reach statistical significance (PERMANOVA, *R*^2^ = 0.2824, *p* = 0.408), the ordination pattern indicated a consistent directional shift of the peanut rhizosphere fungal community in response to intercropping.

**Figure 3:**
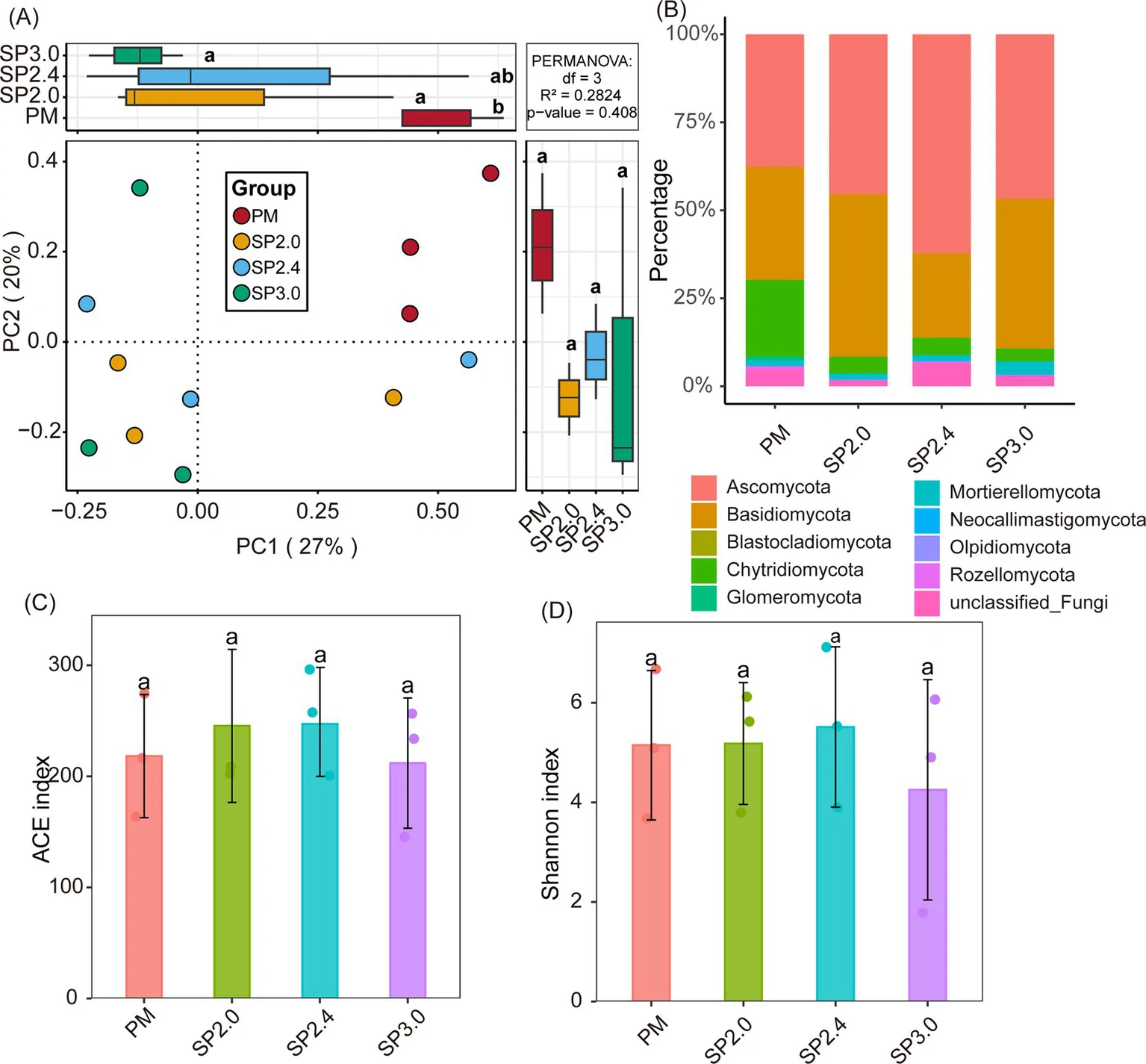
Peanut rhizosphere fungal community composition and diversity under different treatments. **(A)** Principal coordinate analysis (PCoA) based on Bray-Curtis distances, with PERMANOVA statistics; **(B)** Relative abundance of fungal phyla; **(C)** ACE richness index; **(D)** Shannon diversity index. Different letters in **(C,D)** indicate significant differences at *p* < 0.05 (*n* = 3). Each treatment comprised three independent field replicates (*n* = 3), with one composite peanut rhizosphere-soil sample representing each plot.

At the phylum level, Ascomycota dominated the peanut rhizosphere in all treatments, and its relative abundance increased from approximately 38% in PM to 46–62% in the intercropping treatments, with the highest value in SP2.4 (Figure 3B). Basidiomycota accounted for approximately 32% of sequences in PM and increased to approximately 46% in SP2.0 and 43% in SP3.0, but decreased to approximately 24% in SP2.4. In contrast, Chytridiomycota declined sharply from approximately 22% in PM to 4–7% in the intercropping treatments. Mortierellomycota remained a minor component (1–3%) across treatments. At the genus level, community composition also varied among treatments, with treatment-dependent shifts in several abundant or ecologically relevant groups, including Penicillium, Marasmiellus, Aspergillus, Talaromyces, Mortierella, and Trechispora (Supplementary Figure S2). These patterns indicate that the compositional response to intercropping extended below the phylum level.

Alpha diversity indices did not differ significantly among treatments (*p* > 0.05; Figures 3C,D). Nevertheless, the ACE richness index was numerically highest in SP2.0 and SP2.4 (approximately 245 and 247, respectively), intermediate in PM (approximately 220) and lowest in SP3.0 (approximately 212); the Shannon index showed a similar trend. Thus, intercropping did not significantly alter fungal alpha diversity but tended to increase richness at the 2.0 m and 2.4 m spacings.

### 3.3 Intercropping shifts potentially pathogenic and candidate beneficial fungal taxa

DESeq2 differential-abundance analysis identified 28, 37, and 16 fungal taxa with Benjamini–Hochberg FDR-adjusted *p* < 0.05 between PM and SP2.0, SP2.4, and SP3.0, respectively (Figures 4A–C). SP2.4 showed the largest number of differential taxa, spanning Ascomycota, Basidiomycota, Chytridiomycota, Glomeromycota and Mortierellomycota, indicating the strongest restructuring of the peanut rhizosphere mycobiome at the 2.4 m spacing.

**Figure 4:**
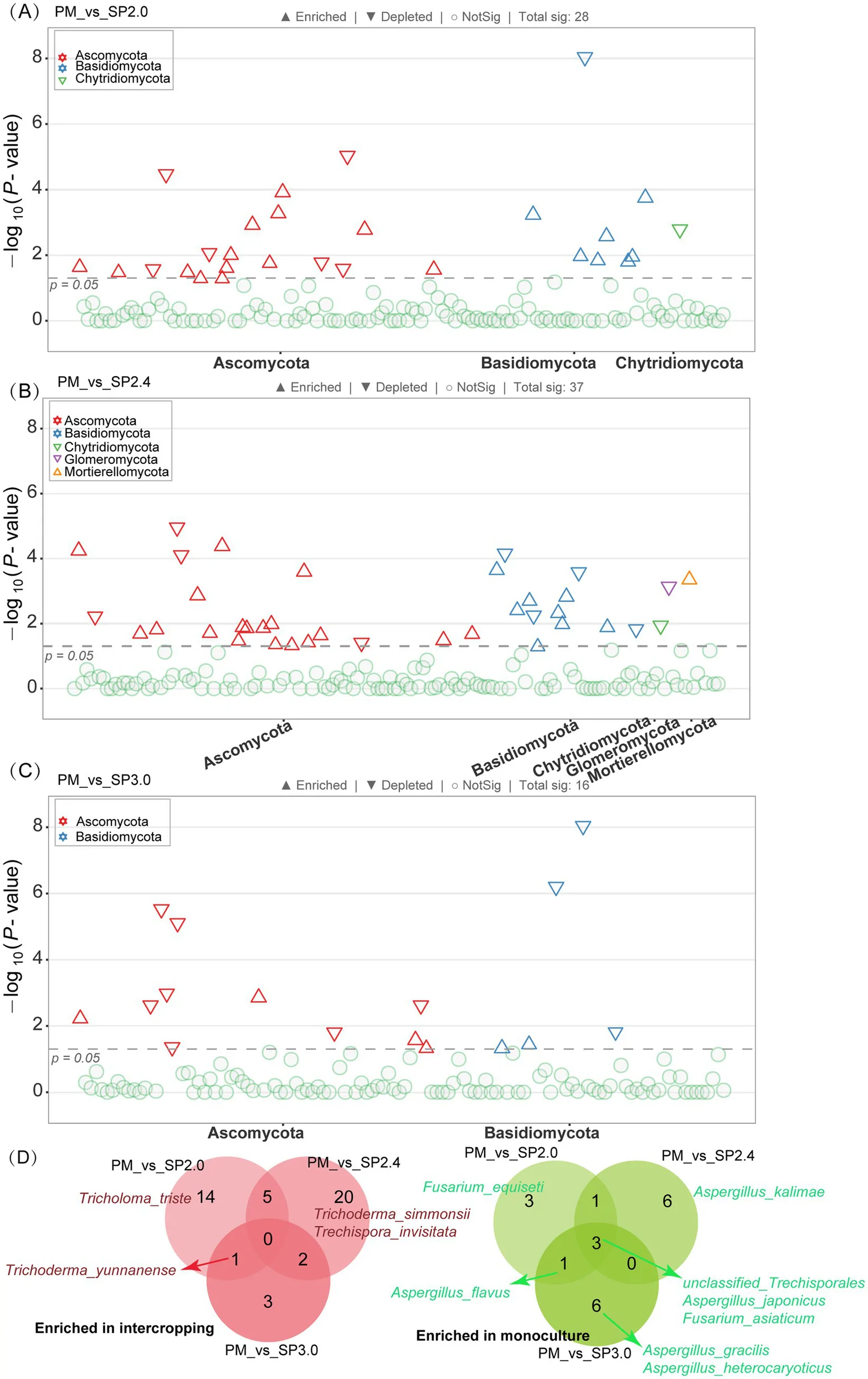
Differential fungal taxa in peanut rhizosphere soil between peanut monoculture and each sugarcane–peanut intercropping treatment. **(A–C)** Manhattan plots showing significantly enriched (upward triangles) and depleted (downward triangles) fungal taxa in SP2.0 **(A)**, SP2.4 **(B)** and SP3.0 **(C)** compared to PM (identified using DESeq2 with Benjamini–Hochberg FDR-adjusted *p* < 0.05). Points are colored by phylum. The numbers of significantly different taxa were 28, 37 and 16 for SP2.0, SP2.4 and SP3.0, respectively. **(D)** Venn diagrams showing shared and unique taxa enriched in intercropping (left) and enriched in monoculture, i.e., depleted by intercropping (right) across the three pairwise comparisons. Key taxa are labeled; species-level labels represent putative ITS1/UNITE taxonomic assignments. The ASV putatively assigned to *Aspergillus flavus* was depleted in both SP2.0 and SP3.0. Each treatment comprised three independent field replicates (*n* = 3), with one composite peanut rhizosphere-soil sample per plot.

Venn analysis of intercropping-enriched taxa revealed that each spacing harbored a distinct set of enriched taxa: 14, 20 and 3 taxa were uniquely enriched in SP2.0, SP2.4 and SP3.0, respectively (Figure 4D, left). An ASV putatively assigned to *Tricholoma triste* was uniquely enriched in SP2.0, whereas ASVs putatively assigned to *Trichoderma simmonsii* and *Trechispora invisitata* were among the taxa uniquely enriched in SP2.4. Notably, an ASV putatively assigned to *Trichoderma yunnanense* was the only taxon shared between SP2.0 and SP3.0, indicating its consistent enrichment at these two spacings.

Conversely, Venn analysis of monoculture-enriched (i.e., intercropping-depleted) taxa identified three taxa—an unclassified Trechisporales, and ASVs putatively assigned to *Aspergillus japonicus*, and *Fusarium asiaticum*—that were depleted by all three intercropping treatments (Figure 4D, right). An ASV putatively assigned to *Aspergillus flavus* was significantly depleted in SP2.0 and SP3.0. In addition, included *F. equiseti*, which was specifically depleted in SP2.0, *Aspergillus kalimae* in SP2.4, and *Aspergillus gracilis* and *Aspergillus heterocaryoticus* in SP3.0. These results show that sugarcane–peanut intercropping reduced several members of the *Aspergillus*/*Fusarium* assemblage in the peanut rhizosphere, although the responsive taxa differed among row spacings. In summary, the main treatment-associated differential taxa were: for SP2.0, depletion of *A. flavus* and *F. equiseti* and enrichment of *T. yunnanense* and *Tricholoma triste*; for SP2.4, enrichment of *T. simmonsii* and *Trechispora invisitata* and depletion of *A. kalimae*; and for SP3.0, depletion of *A. flavus*, *A. gracilis* and *A. heterocaryoticus* and enrichment of *T. yunnanense*.

### 3.4 Intercropping shifts rhizosphere metabolite profiles and enriches candidate bioactive compounds

Untargeted LC–MS/MS metabolomics profiled the peanut rhizosphere metabolome. PCoA based on metabolite abundances showed separation between PM and intercropping treatments along the first two axes, which explained 60 and 15% of the total variation, respectively (PERMANOVA, *R*^2^ = 0.2872, *p* = 0.357; Figure 5A). Metabolite classification revealed that the annotated metabolites were dominated by benzene and substituted derivatives, carboxylic acids and derivatives, fatty acyls, organooxygen compounds and prenol lipids (Figure 5B).

**Figure 5:**
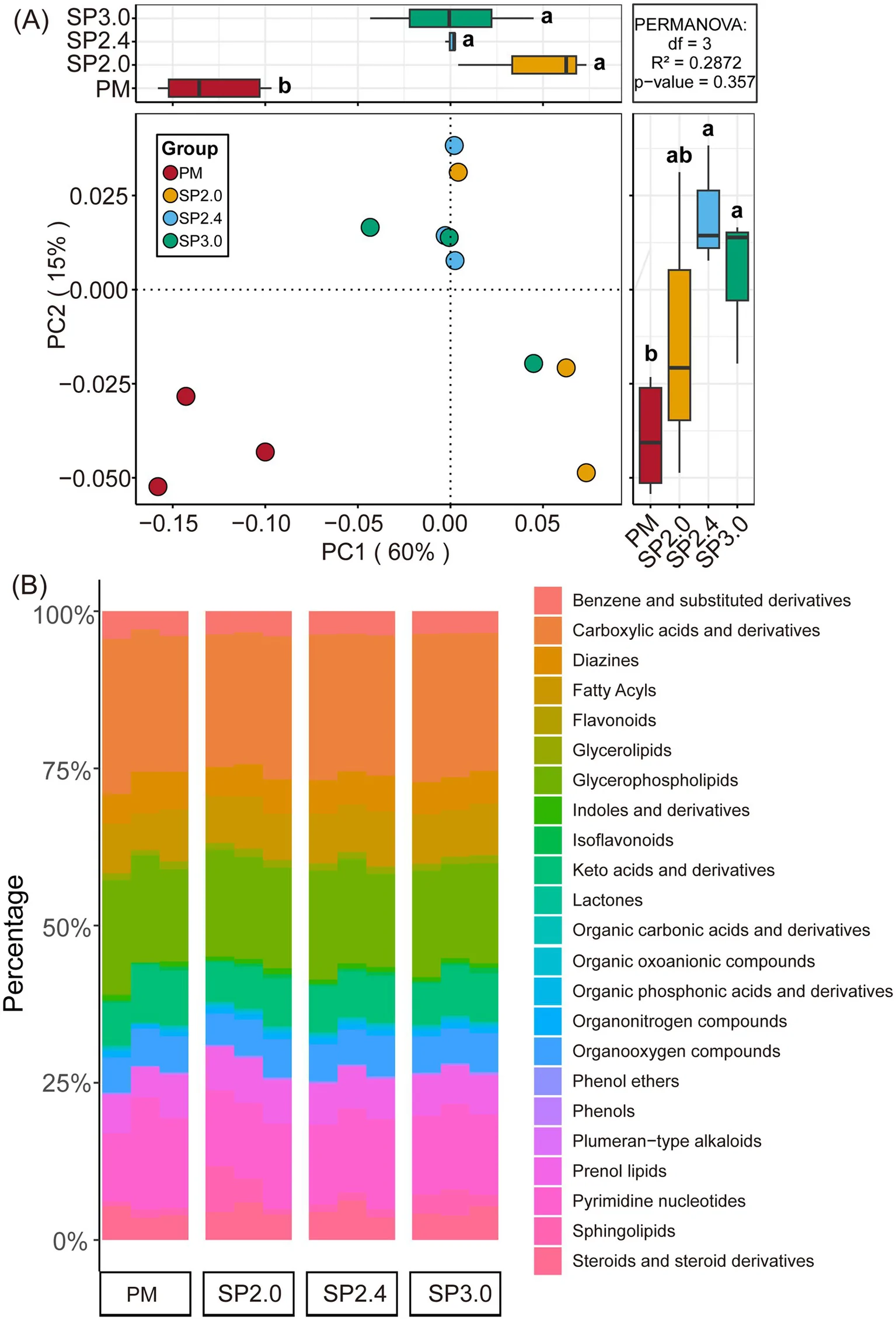
Metabolite profiles of peanut rhizosphere soil under different cropping treatments. **(A)** PCoA based on metabolite abundances, with PERMANOVA statistics; **(B)** Relative abundance of metabolite superclasses. PM = peanut monocropping; SP2.0, SP2.4, SP3.0 = intercropping treatments. Each treatment comprised three independent field replicates (*n* = 3), with one composite peanut rhizosphere-soil sample per plot.

Manhattan plot analysis identified the top differential metabolites in each pairwise comparison (Figures 6A–C). In SP2.0 vs. PM, 4-glucogallic acid showed a log2FC of 7.41 (FDR-adjusted *p* = 0.0047). In SP2.4 vs. PM, cis-resveratrol (log2FC = 2.62, FDR-adjusted *p* = 0.0038), 4-ethyl-2-methoxyphenol (log2FC = 2.36, FDR-adjusted *p* = 0.0102), and 4-hydroxycinnamyl aldehyde (log2FC = 3.59, FDR-adjusted *p* = 0.0305) were among the most responsive features. In SP3.0 vs. PM, vanillic acid showed a log2FC of 1.16 (FDR-adjusted *p* = 0.0410).

**Figure 6:**
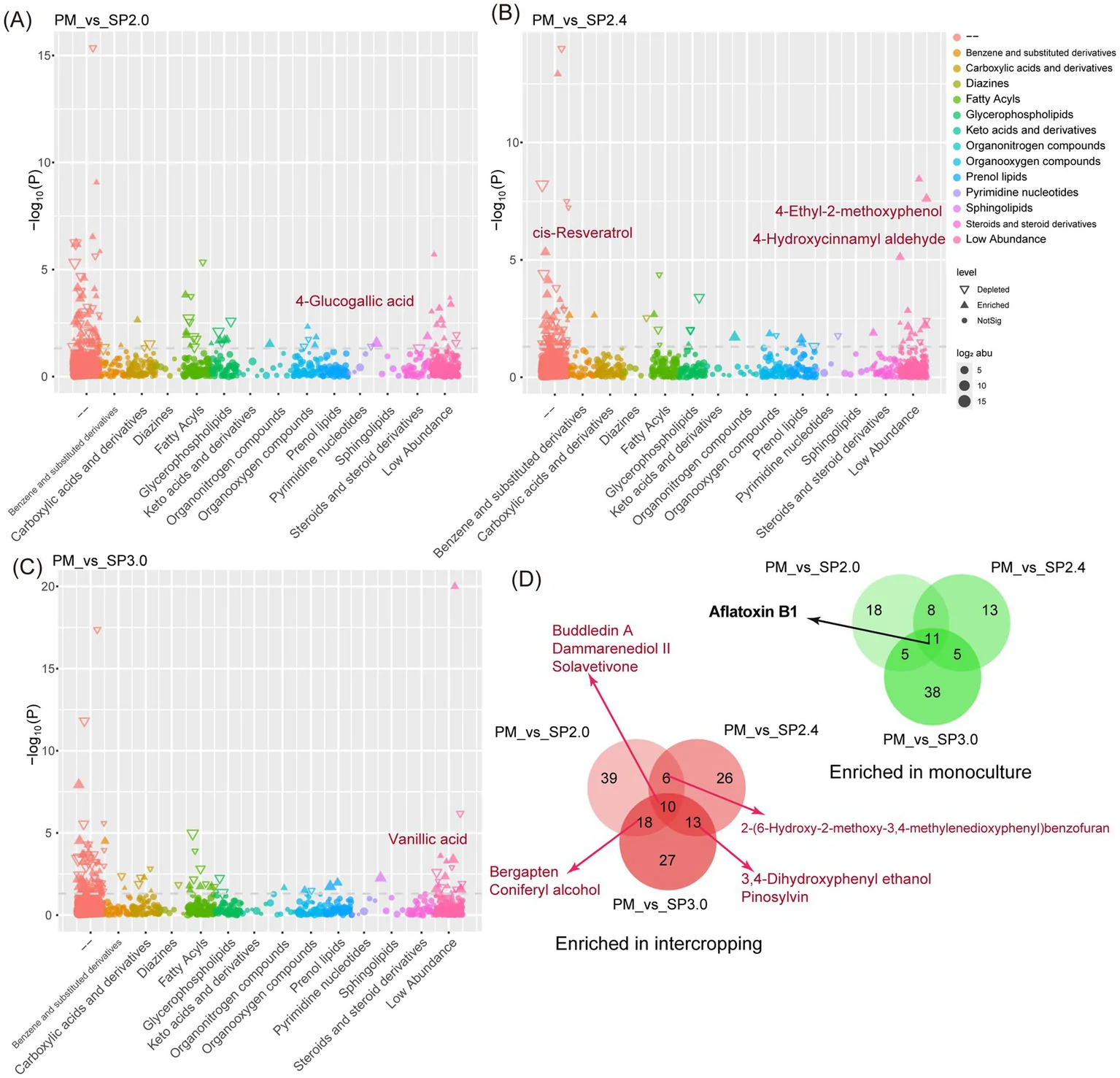
Differentially accumulated metabolite features in peanut rhizosphere soil between peanut monoculture and each sugarcane–peanut intercropping treatment. **(A–C)** Manhattan plots showing significantly enriched (upward triangles) and depleted (downward triangles) metabolites in SP2.0 **(A)**, SP2.4 **(B)** and SP3.0 **(C)** compared to PM. Points are colored by metabolite superclass; the most responsive metabolites are labeled. **(D)** Venn diagrams showing shared and unique metabolites enriched in intercropping (left) and enriched in monoculture (right) across the three pairwise comparisons. A feature putatively annotated as AFB_1_ by untargeted MS2 library matching is indicated among the PM-enriched features shared across all three comparisons. This annotation was not confirmed using an authentic standard; key anti-pathogenic metabolites enriched in intercropping are labeled. Each treatment comprised three independent field replicates (*n* = 3), with one composite peanut rhizosphere-soil sample per plot.

Venn analysis of monoculture-enriched metabolites showed that 18, 13 and 38 metabolites were uniquely enriched in PM relative to SP2.0, SP2.4 and SP3.0, respectively, and 11 metabolites were shared by all three comparisons (Figure 6D, right). Among the shared PM-enriched features, one LC–MS feature was putatively annotated as AFB_1_ based on the original untargeted MS2 library-matching workflow. Because this annotation was not confirmed using an authentic standard, it should be interpreted as tentative and does not establish AFB_1_ concentration or contamination in the rhizosphere samples.

Venn analysis of intercropping-enriched metabolite features identified 39, 26, and 27 features uniquely enriched in SP2.0, SP2.4, and SP3.0, respectively, six shared between SP2.0 and SP2.4, 18 shared between SP2.0 and SP3.0, 13 shared between SP2.4 and SP3.0, and ten shared across all three intercropping treatments (Figure 6D, left). Several shared features were annotated as compounds with reported roles in plant defense, antioxidant activity, or fungal inhibition: bergapten and coniferyl alcohol were shared between SP2.0 and SP3.0; 3,4-dihydroxyphenyl ethanol (hydroxytyrosol) and pinosylvin were shared between SP2.4 and SP3.0; 2-(6-hydroxy-2-methoxy-3,4-methylenedioxyphenyl)benzofuran was shared between SP2.0 and SP2.4; and the terpenoids Buddledin A, Dammarenediol II, and solavetivone were shared across all three intercropping treatments (Table 2).

**Table 2:** Candidate bioactive metabolites enriched in intercropping treatments and their reported activities.

Metabolite	Class	Enriched in	Reported activity	Reference
Pinosylvin	Stilbenoid	SP2.4 ∩ SP3.0	Reported antifungal activity	Lee et al. (2005)
Cis-resveratrol	Stilbenoid	SP2.4	Reported antifungal activity; synergistic antifungal activity with azoles against *Candida albicans*	Wang et al. (2021)
Coniferyl alcohol	Phenylpropanoid	SP2.0 ∩ SP3.0	Phenylpropanoid with reported antifungal activity and potential involvement in plant defense responses	Keen and Littlefield (1979)
Vanillic acid	Phenolic acid	SP3.0	Reported antifungal activity against *Aspergillus flavus*	Kim et al. (2005)
3,4-Dihydroxyphenyl	Phenolic	SP2.4 ∩ SP3.0	Antioxidant compound with reported broad-spectrum antifungal activity *in vitro*	Zorić et al. (2013)
Bergapten	Furanocoumarin	SP2.0 ∩ SP3.0	Furanocoumarin with reported antimicrobial and antifungal activity	Özçelik et al. (2004)
2-(6-Hydroxy-2-methoxy- 3,4-methylenedioxy-phenyl)benzofuran	Benzofuran	SP2.0 ∩ SP2.4	Yeast-associated benzofuran with reported antioxidant activity	McKittrick and Stevenson (1984)
Solavetivone	Sesquiterpenoid	All three	Sesquiterpenoid phytoalexin with reported antifungal activity	Harris and Dennis (1976)
Buddledin A	Sesquiterpenoid	All three	Reported antifungal activity against dermatophytic fungi	Mensah et al. (2000)
Dammarenediol II	Triterpenoid	All three	Intermediate in the biosynthesis of dammarane-type ginsenosides	Tansakul et al. (2006)

“Enriched in” indicates the pairwise comparisons (each intercropping treatment vs. PM) in which the metabolite was significantly enriched according to the DAM criteria of |log2FC| ≥ 1 and Benjamini–Hochberg FDR-adjusted *p* < 0.05, based on the Venn analysis in Figure 6D.

### 3.5 Row spacing determines the strength of the intercropping effect

Comparison across the three row spacings showed that the intercropping effect was spacing-dependent (Table 1; Figures 2–6). For fungal taxa reported at species level below, names refer to putative ITS1/UNITE assignments. SP2.0 achieved the highest total LER (1.48) and sugarcane partial LER (0.96), the longest lateral branches, significant depletion of *A. flavus*, enrichment of *T. yunnanense* and *Tricholoma triste*, and the highest linoleic acid content (together with SP2.4). SP2.4 showed the strongest rhizosphere restructuring, with the largest number of differential fungal taxa (37) and the enrichment of *Trichoderma simmonsii* and *Trechispora invisitata*, together with cis-resveratrol, 4-ethyl-2-methoxyphenol and 4-hydroxycinnamyl aldehyde; kernels from SP2.4 also had the highest crude protein content (together with SP3.0) and a crude fat content comparable to PM. SP3.0 maintained the highest peanut yield and peanut partial LER (0.77) among the intercropping treatments and also depleted *A. flavus* and enriched *T. yunnanense* and vanillic acid, but showed the fewest differential fungal taxa (16) and the lowest sugarcane yield. Taken together, 2.0–2.4 m constitutes the optimal row-spacing window, with 2.0 m maximizing land productivity and 2.4 m maximizing rhizosphere biocontrol restructuring and kernel protein quality.

## 4 Discussion

### 4.1 Sugarcane–peanut intercropping improves land productivity

Although peanut yield per unit land area was lower under intercropping than under peanut monoculture, this reduction should be interpreted in the context of the lower peanut planting density and the simultaneous production of sugarcane. Total LER exceeded 1 in all three intercropping treatments, and SP2.0 achieved the highest total LER of 1.48 (Table 1; Figure 2). This result indicates that less land would be required under intercropping than under the corresponding monocultures to produce an equivalent combined yield. A meta-analysis of annual intercropping systems reported an average LER of 1.22 and showed that temporal niche differentiation can contribute substantially to the land-use advantage of intercrops, particularly in systems combining C3 and C4 species (Yu et al., 2015). In cereal–legume systems, belowground interspecific interactions and root-derived signaling compounds can enhance legume nodulation and biological nitrogen fixation, thereby improving nitrogen acquisition at the cropping-system level (Li et al., 2016). Sugarcane–legume intercropping can also change rhizosphere microbial abundance and soil nutrient conditions, potentially facilitating nutrient mobilization and acquisition by the component crops (Tang et al., 2021; Pang et al., 2022; Tang et al., 2022). Together, these findings suggest that root-exudate-mediated plant–plant and plant–microbe communication may contribute to complementary resource acquisition in sugarcane–peanut intercropping, although nodulation and biological N_2_ fixation were not directly measured in the present study. These processes provide plausible explanations for the LER advantage observed in the present experiment.

The crop-level origin of the land-use advantage varied among row spacings. SP2.0 had the highest sugarcane partial LER and total LER, suggesting that the relatively narrow configuration favored system productivity mainly by maintaining the contribution of sugarcane. By contrast, SP3.0 had the highest peanut yield and peanut partial LER among the intercropping treatments but comparatively low sugarcane yield and sugarcane partial LER. SP2.4 showed an intermediate and more balanced yield structure (Table 1; Figure 2). Increasing row spacing therefore appeared to reduce the competitive pressure experienced by peanut while decreasing the contribution of sugarcane to total productivity. These contrasting responses indicate that row spacing regulated the balance between interspecific competition and facilitation rather than producing a simple linear response.

The observed row-spacing effects may also be related to changes in the size, intensity, and spatial location of the root-interaction zone. Studies in sugarcane–peanut systems have shown that intercropping can alter root-exuded organic acids, soluble sugars, phenolic acids, rhizosphere enzyme activities, and soil nutrient availability, and that these effects vary according to crop species and developmental stage (Tang et al., 2022; Tang et al., 2024). Intercropping has also been associated with changes in rhizosphere purine metabolism and metabolites related to soil phosphorus and potassium availability (Tang et al., 2022). Therefore, spatial configuration may affect crop performance by changing not only aboveground competition but also the intensity of belowground chemical and microbial interactions.

Intercropping also affected peanut kernel composition. SP2.4 and SP3.0 increased crude protein content, whereas SP2.0 and SP2.4 maintained relatively high linoleic acid contents. Crude fat was lowest in SP2.0, while SP2.4 maintained a crude fat content similar to that of PM (Table 1; Figure 2). The simultaneous increase in crude protein and maintenance of crude fat in SP2.4 suggest that this configuration may provide a favorable balance between nutritional and oil-processing traits. Nevertheless, peanut protein, total oil, and fatty-acid composition are controlled by genotype, seed developmental stage, temperature, water availability, and genotype-by-environment interactions (Wang et al., 2018; Liu and Li, 2023; Wang et al., 2024). These quality changes may result from intercropping-induced shifts in carbon–nitrogen balance, microclimate, nutrient acquisition, or seed filling, but further physiological and molecular analyses are needed to clarify the underlying mechanisms.

### 4.2 Intercropping selectively restructures the fungal community

Ascomycota remained the dominant fungal phylum in all treatments and increased from approximately 38% in PM to 46–62% in the intercropping treatments. Chytridiomycota declined markedly under intercropping, whereas Basidiomycota increased in SP2.0 and SP3.0 but decreased in SP2.4 (Figure 3B). These nonuniform responses indicate that intercropping imposed selective pressures on particular fungal groups rather than causing a consistent increase or decrease across the entire fungal community. Similar context-dependent shifts in fungal composition and functional guilds have been observed in sugarcane–soybean intercropping (Lian et al., 2018). Because fungal communities are influenced by crop, soil, developmental, and management factors, phylum-level changes should not be interpreted as inherently beneficial or detrimental.

The absence of significant differences in fungal alpha diversity suggests that intercropping did not improve rhizosphere microecology through a broad increase in richness or evenness (Figures 3C,D). Instead, functional improvement may depend on the recruitment or depletion of particular taxa and their interactions rather than on overall microbial diversity (Mendes et al., 2011; Yang et al., 2015; Zhou et al., 2023). This interpretation is supported by the 28, 37, and 16 differentially abundant fungal taxa detected in SP2.0, SP2.4, and SP3.0, respectively, including shifts in *Aspergillus*, *Fusarium*, and *Trichoderma* (Figures 4A–C). The slightly higher ACE and Shannon values in SP2.0 and SP2.4 should therefore be regarded as numerical trends, while the principal fungal response appears to involve selective turnover among functional groups.

### 4.3 Intercropping shifts potentially pathogenic and candidate beneficial fungal assemblages

A notable finding was the significant reduction of an ASV putatively assigned to *A. flavus* in SP2.0 and SP3.0 (Figures 4A,C,D), together with depletion of several other *Aspergillus* and *Fusarium* taxa. These genera contain important soilborne pathogens, but amplicon-based relative abundance alone does not establish pathogenic activity, strain-level toxigenicity, or effects on harvested kernels. Populations of *A. flavus* include both toxigenic and non-toxigenic strains that differ in secondary-metabolite pathways and ecological behavior (Hulikunte Mallikarjunaiah et al., 2017). The observed decrease should therefore be interpreted primarily as evidence that intercropping altered the composition of a potentially pathogenic rhizosphere fungal assemblage.

Despite the feature putatively annotated as AFB_1_ showed very low intensity in SP2.4, the ASV putatively assigned to *A. flavus* was not significantly depleted relative to the corresponding monoculture, further supporting the view that intercropping affected fungal toxigenicity as well as abundance (Figures 4B, 6D). SP2.4 may have reduced toxin production per unit fungal biomass, shifted the balance between toxigenic and atoxigenic genotypes, altered expression of the aflatoxin gene cluster, or promoted conditions that accelerated toxin transformation or degradation. Environmental conditions, temperature, and fungal oxidative status can substantially alter aflatoxin-pathway expression and secondary metabolism in *A. flavus* (Bai et al., 2015; Wang et al., 2015; Fountain et al., 2018).

ASVs putatively assigned to *Aspergillus japonicus* and *Fusarium asiaticum* were depleted under all three intercropping configurations. In addition, ASVs assigned putatively to *F. equiseti* was specifically depleted in SP2.0, *A. kalimae* in SP2.4, and *A. gracilis* and *A. heterocaryoticus* in SP3.0 (Figures 4A–D). The simultaneous depletion of several Aspergillus and Fusarium taxa across the intercropping treatments indicates that the fungal response involved multiple members of this potentially pathogenic assemblage rather than an isolated response by a single taxon (Figures 4A–D). The simultaneous depletion of multiple members under intercropping is therefore more informative for evaluating rhizosphere microecological change than any single taxon or metabolite feature.

Intercropping simultaneously enriched fungi with potential antagonistic or plant-beneficial functions. An ASV putatively assigned to *Trichoderma yunnanense* was enriched in SP2.0 and SP3.0, whereas ASVs putatively assigned to *T. simmonsii* and *Trechispora invisitata* were enriched in SP2.4 (Figures 4A–D). The enrichment of these candidate beneficial taxa is biologically plausible because *Trichoderma* isolates can suppress competing fungi through rapid nutrient capture, mycoparasitism, secretion of cell-wall-degrading enzymes, and production of diffusible or volatile metabolites (Harman et al., 2004; Saravanakumar et al., 2017). Nevertheless, the ecological functions of the specific taxa detected here require isolation-based antagonism assays and absolute quantification. We therefore describe them as candidate beneficial fungi rather than confirmed biocontrol agents.

### 4.4 Intercropping-associated metabolites may contribute to rhizosphere niche regulation

The metabolite PCoA showed a tendency for PM to separate from the intercropping treatments, although the overall treatment effect was not significant (Figure 5A). The annotated features were dominated by benzene and substituted derivatives, carboxylic acids and derivatives, fatty acyls, organooxygen compounds, and prenol lipids (Figure 5B). The most responsive features varied among row spacings: 4-glucogallic acid was prominent in SP2.0; cis-resveratrol, 4-ethyl-2-methoxyphenol, and 4-hydroxycinnamyl aldehyde were prominent in SP2.4; and vanillic acid was prominent in SP3.0 (Figures 6A–C). These phenolic and stilbenoid compounds have reported roles in plant defense, antioxidant regulation, or fungal inhibition (Kim et al., 2005; Wang et al., 2015). Their enrichment is therefore consistent with a chemically mediated shift in rhizosphere niche conditions, but their origin, *in situ* concentration, and direct ecological effects were not determined in this study.

Vanillic acid, rutin, and alpha-tocopherol were negatively associated with the ASV putatively assigned to *A. flavus*, while several antioxidant-related features showed opposing associations with the *Aspergillus*/*Fusarium*-dominated network module (Figure 6; Table 2). Oxidative status can influence fungal development and secondary metabolism, providing a plausible biological context for these associations (Caceres et al., 2017; Fountain et al., 2018). However, correlation does not demonstrate direct inhibition, and untargeted signal intensity does not establish bioactive concentration. Targeted metabolite quantification and fungal bioassays are needed to test whether these compounds contribute causally to the observed community shifts. Structural equation modeling may be useful for evaluating hypothesized pathways in future studies with substantially greater replication, together with targeted and manipulative experiments.

The intercropping treatments also shared several enriched metabolites. Bergapten and coniferyl alcohol were shared between SP2.0 and SP3.0; 3,4-dihydroxyphenyl ethanol and pinosylvin were shared between SP2.4 and SP3.0; and Buddledin A, Dammarenediol II, and solavetivone were enriched across all three intercropping treatments (Figure 6D; Table 2). These compounds may represent additional components of plant chemical defense or microbial metabolism. The biological origin of these metabolites is also unresolved. They may have been released directly by peanut or sugarcane roots, produced during plant defense, transformed from plant precursors by microorganisms, or synthesized by the rhizosphere microbiota. Sugarcane–peanut intercropping has previously been shown to increase phenolic acids, organic acids, and soluble sugars in peanut root exudates and to change both root and rhizosphere-soil metabolite profiles (Tang et al., 2022; Tang et al., 2024). In other intercropping systems, root exudates from one crop have altered microbial recruitment and disease suppression in the neighboring crop (Li et al., 2016; Zhou et al., 2023). These findings provide a plausible context for root-exudate-mediated plant–plant and plant–microbe communication in intercropping systems, although the origin and signaling functions of the metabolites detected in the present study were not directly determined.

### 4.5 Integration with sustainable farming systems

Overall, the intercropping benefit was spacing-dependent, with 2.0–2.4 m forming the most favorable window through partly different mechanisms. At 2.0 m, close sugarcane–peanut proximity produced the highest total LER (1.48) and sugarcane partial LER (0.96), depletion of several potentially pathogenic taxa, enrichment of an ASV putatively assigned to *T. yunnanense*, and high kernel linoleic acid content. At 2.4 m, the rhizosphere showed the most extensive fungal restructuring (37 differential taxa), enrichment of ASVs putatively assigned to *T. simmonsii* and *Trechispora invisitata*, accumulation of cis-resveratrol and other defense-related metabolites, and high kernel crude protein content. At 3.0 m, weaker interspecific interaction preserved peanut yield but produced fewer fungal changes and the lowest sugarcane yield.

From an agroecosystem perspective, these belowground shifts may have functional consequences beyond changes in taxonomic composition alone. The reduced representation of potentially pathogenic *Aspergillus*/*Fusarium* taxa, together with enrichment of candidate beneficial fungi and defense-associated metabolites, is consistent with a rhizosphere state potentially less conducive to pathogen proliferation and more supportive of crop performance. Importantly, these belowground responses occurred alongside total LER values > 1 in all intercropping treatments. The contrasting responses among row spacings further suggest a trade-off between belowground ecological restructuring and crop productivity: SP2.0 achieved the highest total LER, whereas SP2.4 showed the most extensive fungal and metabolic reorganization while maintaining favorable kernel-quality traits. However, because nutrient fluxes, pathogen activity, microbial functional genes, and other ecosystem processes were not directly quantified, these functional implications should be regarded as hypotheses requiring further validation.

The concurrent treatment-related shifts in potentially pathogenic fungi, candidate beneficial taxa, and defense-related metabolite features are relevant to the broader goals of sustainable agriculture. These patterns suggest that sugarcane–peanut intercropping may promote a rhizosphere state associated with reduced representation of potentially harmful fungal taxa while maintaining or improving cropping-system productivity. This biological control approach is particularly relevant for smallholder farmers in tropical and subtropical regions where aflatoxin contamination poses serious health and economic risks (Wu and Khlangwiset, 2010).

The treatment-associated fungal and metabolite features identified here represent ecological signatures rather than a defined microbial intervention. In parallel, our group has developed the synthetic microbial community ARC, a four-strain non-rhizobial bacterial consortium selected to suppress *Aspergillus flavus* while remaining compatible with Bradyrhizobium. Earlier field demonstrations of the ARC-BBBE inoculant reported enhanced peanut nodulation, nitrogenase activity and yield together with reduced rhizosphere *A. flavus*, whereas subsequent multi-site work with SynCom ARC further showed coupled aflatoxin suppression and promotion of rhizobial nodulation and nitrogen fixation (Zhou et al., 2022; Ji et al., 2023; Zhang et al., 2026). These studies demonstrate the potential of microbiome engineering to coordinate pathogen suppression with legume symbiosis. The treatment-associated fungal and metabolite patterns observed here extend this perspective to field-scale crop diversification and provide ecological insights for microbiome-informed management. ARC therefore offers a practical translational model for converting field-derived ecological knowledge into targeted microbial interventions. Integrating intercropping with defined communities such as ARC may provide a promising strategy for combining natural rhizosphere regulation with designed microbial functions, thereby supporting the development of more productive, resilient and sustainable peanut-based cropping systems.

Furthermore, changes in peanut kernel quality traits—higher crude protein at 2.4–3.0 m and relatively high linoleic acid at 2.0–2.4 m—show that the agronomic consequences of intercropping extend beyond land-use efficiency. Considered together with the rhizosphere fungal and metabolic responses, these results make intercropping a promising strategy for sustainable intensification of long-term peanut production systems.

## 5 Limitations and future directions

This study provides a framework for understanding how sugarcane–peanut intercropping coordinates rhizosphere fungal communities, metabolite profiles, and land-use efficiency in a long-term monocropped peanut field. Several limitations should guide interpretation. First, the experiment was conducted at a single site during one growing season with three field replicates per treatment. Second, fungal data were based on relative ITS1 amplicon abundance, and ITS1 does not provide uniform species-level resolution across all fungal lineages. Accordingly, species-level names reported in this study should be regarded as putative taxonomic assignments rather than definitive identifications. The amplicon data also did not resolve strain-level function or absolute population size. Third, metabolite identities were assigned by untargeted library matching without authentic-standard confirmation; in particular, AFB_1_ content was not directly measured, and the feature putatively annotated as AFB_1_ cannot establish AFB_1_ concentration, contamination, or food-safety outcomes. Future work should combine multi-year and multi-location trials with soil physicochemical measurements, absolute quantification of key fungal taxa, targeted metabolomics, isotope tracing, and manipulation experiments. If aflatoxin risk is to be evaluated, validated targeted LC–MS/MS quantification should be performed independently in rhizosphere soil and harvested kernels. A factorial experiment crossing intercropping configuration with ARC inoculation would provide a direct test of whether crop diversification and defined microbiome engineering act additively or interactively. Such experiments should quantify ARC strain persistence, absolute *A. flavus* and Bradyrhizobium abundance, nodulation, nitrogenase activity, targeted AFB_1_ concentrations in rhizosphere soil and harvested kernels, and plot-level crop productivity.

## 6 Conclusion

This study demonstrates coordinated, row-spacing-dependent changes in land productivity, peanut quality, rhizosphere fungal communities, and metabolite profiles under sugarcane–peanut intercropping. Intercropping depleted several potentially pathogenic *Aspergillus* and *Fusarium* taxa, enriched candidate beneficial fungi such as *Trichoderma*, and promoted phenolic, stilbenoid, terpenoid, and antioxidant-related metabolite features. These responses indicate that intercropping was accompanied by parallel treatment-associated changes in rhizosphere fungal community composition and metabolite profiles in a long-term monocropped peanut field. Spatial configuration regulated the balance between crop productivity and belowground ecological responses: SP2.0 maximized land-use efficiency, whereas SP2.4 produced the most extensive fungal and metabolic reorganization while maintaining favorable kernel-quality traits. Because the study was conducted at one site with three field replicates per treatment, and because the functional relationships between the fungal and metabolite responses were not directly tested, the proposed ecological mechanisms require further validation. Further validation across multiple soils, climates, years, and crop genotypes, together with functional experiments and targeted quantification of AFB_1_ in both rhizosphere soil and harvested kernels, will be necessary to establish the robustness and food-safety relevance of this intercropping strategy.

## Data Availability

The datasets presented in this study can be found in online repositories. The raw ITS amplicon sequencing data have been deposited in the NCBI Sequence Read Archive (SRA) under BioProject accession number PRJNA1534510 (https://www.ncbi.nlm.nih.gov/bioproject/PRJNA1534510). The differential metabolites identified in this study are provided in the Supplementary material. All other data are available from the corresponding authors upon reasonable request.
