# Supplementary material for: Sugarcane–peanut intercropping improves rhizosphere fungal communities and metabolites following long-term continuous peanut monoculture
Source: Front Microbiol. 2026 Oct 23;17:1957778. doi: 10.3389/fmicb.2026.1957778 (PMC13739793; doi:10.3389/fmicb.2026.1957778)
Supplement: Supplementary file 1 [file Data_Sheet_1.docx]

Supplementary Material

# 1 Supplementary Figures and Tables

## 1.1 Supplementary Figures

**Supplementary Figure S1.** **Rarefaction curves and Shannon curves of peanut rhizosphere fungal communities under monocropping and sugarcane-peanut intercropping treatments.** All curves were generated by taking the number of observed ASVs and Shannon index values as the response variables against gradient sequencing depth for each peanut cultivation group (PM, SP2.0, SP2.4, and SP3.0). Shaded areas indicate standard error across biological replicates. All curves approached clear saturation plateaus, indicating sufficient sequencing depth to capture the majority of fungi diversity across samples and supporting high sequencing completeness.

Supplementary Figure S2. Genus-level composition of peanut rhizosphere fungal communities under different cropping treatments. Stacked bars show the mean relative abundances of the most abundant genus-level fungal taxonomic groups in each treatment (n = 3 independent field plots per treatment), with less abundant taxa grouped as “Others”. PM = peanut monocropping; SP2.0, SP2.4, and SP3.0 = sugarcane–peanut intercropping with sugarcane row spacings of 2.0, 2.4, and 3.0 m, respectively.

## 1.2 Supplementary Tables

**Supplementary Table S1.** Sample-level sequencing quality-control statistics and observed fungal ASV richness.

| **Treatment** | **Sample ID** | **Raw reads** | **Clean reads** | **Denoised reads** | **Merged reads** | **Non-chimeric reads** | **Rarefied fungal reads** | **Observed ASVs** |
| --- | --- | --- | --- | --- | --- | --- | --- | --- |
| PM | M12T1 | 80,227 | 80,020 | 79,961 | 78,085 | 77,664 | 73,394 | 200 |
| PM | M12T2 | 80,350 | 80,036 | 80,020 | 77,632 | 77,258 | 73,394 | 161 |
| PM | M12T3 | 79,761 | 79,423 | 79,415 | 77,798 | 77,746 | 73,394 | 193 |
| SP2.0 | I20T1 | 79,753 | 79,486 | 79,422 | 78,251 | 78,015 | 73,394 | 191 |
| SP2.0 | I20T2 | 79,856 | 79,580 | 79,524 | 77,463 | 77,378 | 73,394 | 226 |
| SP2.0 | I20T3 | 79,956 | 79,671 | 79,638 | 77,207 | 77,087 | 73,394 | 157 |
| SP2.4 | I24T1 | 80,004 | 79,741 | 79,708 | 78,290 | 77,648 | 73,394 | 217 |
| SP2.4 | I24T2 | 80,170 | 79,824 | 79,802 | 78,248 | 78,212 | 73,394 | 305 |
| SP2.4 | I24T3 | 79,974 | 79,617 | 79,579 | 77,519 | 77,488 | 73,394 | 164 |
| SP3.0 | I30T1 | 79,661 | 79,338 | 79,310 | 77,570 | 77,315 | 73,394 | 184 |
| SP3.0 | I30T2 | 79,875 | 79,608 | 79,539 | 77,976 | 77,929 | 73,394 | 110 |
| SP3.0 | I30T3 | 79,767 | 79,436 | 79,404 | 77,823 | 76,264 | 73,394 | 234 |

Note: Taxonomic assignment was performed against the UNITE fungal database. Only ASVs assigned to the kingdom Fungi were retained for downstream fungal community analyses. The fungal feature table was rarefied to 73,394 sequences per sample before calculation of observed ASV richness. PM = peanut monocropping; SP2.0, SP2.4, and SP3.0 = sugarcane–peanut intercropping with sugarcane row spacings of 2.0, 2.4, and 3.0 m, respectively.
